# Monolayer-directed Assembly and Magnetic Properties of FePt Nanoparticles on Patterned Aluminum Oxide

**DOI:** 10.3390/iijms11031162

**Published:** 2010-03-19

**Authors:** Oktay Yildirim, Tian Gang, Sachin Kinge, David N. Reinhoudt, Dave H.A. Blank, Wilfred G. van der Wiel, Guus Rijnders, Jurriaan Huskens

**Affiliations:** 1 Molecular Nanofabrication Group, MESA+ Institute for Nanotechnology, University of Twente, P.O. Box 217, 7500 AE, Enschede, The Netherlands; 2 Inorganic Materials Science, MESA+ Institute for Nanotechnology, University of Twente, P.O. Box 217, 7500 AE, Enschede, The Netherlands; 3 NanoElectronics Group, MESA+ Institute for Nanotechnology, University of Twente, P.O. Box 217, 7500 AE, Enschede, The Netherlands; 4 Supramolecular Chemistry & Technology, MESA+ Institute for Nanotechnology, University of Twente, P.O. Box 217, 7500 AE, Enschede, The Netherlands

**Keywords:** SAM, Al_2_O_3_, FePt, ferromagnetic, nanoparticle

## Abstract

FePt nanoparticles (NPs) were assembled on aluminum oxide substrates, and their ferromagnetic properties were studied before and after thermal annealing. For the first time, phosph(on)ates were used as an adsorbate to form self-assembled monolayers (SAMs) on alumina to direct the assembly of NPs onto the surface. The Al_2_O_3_ substrates were functionalized with aminobutylphosphonic acid (ABP) or phosphonoundecanoic acid (PNDA) SAMs or with poly(ethyleneimine) (PEI) as a reference. FePt NPs assembled on all of these monolayers, but much less on unmodified Al_2_O_3_, which shows that ligand exchange at the NPs is the most likely mechanism of attachment. Proper modification of the Al_2_O_3_ surface and controlling the immersion time of the modified Al_2_O_3_ substrates into the FePt NP solution resulted in FePt NPs assembly with controlled NP density. Alumina substrates were patterned by microcontact printing using aminobutylphosphonic acid as the ink, allowing local NP assembly. Thermal annealing under reducing conditions (96%N_2_/4%H_2_) led to a phase change of the FePt NPs from the disordered FCC phase to the ordered FCT phase. This resulted in ferromagnetic behavior at room temperature. Such a process can potentially be applied in the fabrication of spintronic devices.

## 1. Introduction

Recently, ferromagnetic nanoparticles (FePt NPs) have attracted interest due to their high chemical stability, magnetic properties and small size. This renders them potential candidates for application in spintronic devices, magnetic sensing and ultra-high density data storage [1–14]. FePt NPs have a high magnetocrystalline anisotropy (10^8^ erg/cm^3^), which should allow the use of small, thermally stable magnetic grains [1–7]. In addition, FePt NPs have a higher chemical stability than other hard magnetic materials [1]. Their well-defined boundaries and small size are very suitable to reach ultra-high storage densities with reduced noise [2,15] in the order of terabit/inch^2^. For the use of FePt NPs in magnetic applications, it is necessary to have a well-controlled assembly process and to cover a sizeable area with high packing density [1,2,10,13]. One method used to attach the particles on the surface is by self-assembly with the help of a coupling layer such as poly (ethyleneimine) (PEI) [1] or an aminosilane [13].

Metal oxides have interesting electronic, optical and magnetic properties and can be insulating, semiconducting, metallic, superconducting, ferroelectric, piezoelectric, ferromagnetic, non-linear optic, colossal magnetoresistant, *etc.* [16–19]. They can be grown epitaxially by pulsed laser deposition to have well controlled interfaces [18,20–23]. So far, all studies on the assembly of FePt NPs have focused on SiO_2_ substrates [1–5,8–10,13,24]. However, for application of nanoparticles in spintronic devices, Al_2_O_3_ is an important substrate because it is the dielectric material of choice in electronic device fabrication [25] and the most used dielectric in magnetic tunneling junctions (MTJs) [26–30]. The latter consist of two ferromagnetic electrodes separated by an insulating barrier, and they are promising candidates for spintronic devices, in which signal detection is achieved via tunneling magnetoresistance (TMR) [27]. The surface properties of Al_2_O_3_ can easily be changed by annealing to get an ultra-smooth surface [31–32].

Self-assembled monolayers (SAMs), in particular thiols on gold and silanes on SiO_2_, have been studied extensively [33–34], but SAMs on metal oxides are relatively new. Alkyl phosphates and alkyl phosphonates form SAMs with high ambient stability on metal oxides such as Ta_2_O_5_, Al_2_O_3_, ZrO_2_ and TiO_2_ without the need for controlled environmental conditions [25,31,35–40].

In this paper, we show the assembly of FePt NPs on Al_2_O_3_ substrates via ligand exchange on SAMs of PEI, aminobutylphosphonic acid (ABP), or phosphonoundecanoic acid (PNDA). The adsorbate molecules are used to direct the assembly of the FePt NPs on the alumina surface. The FePt coverage is controlled by the surface functionalization and by change of the immersion time. The NPs are assembled onto patterned regions of the substrate by employing microcontact printing. Thermal annealing is used to achieve a phase transition of the FePt NPs and to provide ferromagnetic behavior at room temperature [1]. Ferromagnetic properties of the NPs are addressed by measuring the magnetic moment (M) as a function of the strength of an applied magnetic field, with a vibrating sample magnetometer (VSM) before and after thermal annealing.

## 2. Results and Discussion

**Figure 1.**
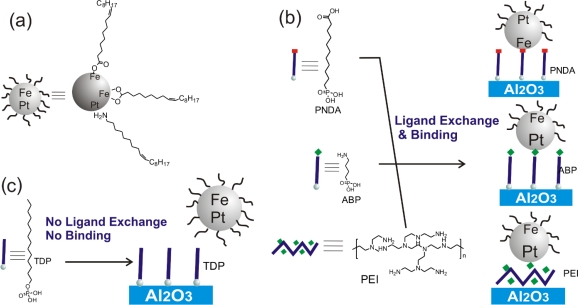
**(a)** FePt NPs stabilized with oleic acid and oleyl amine. **(b)** Adsorption of FePt NPs occurs through ligand exchange onto amino (aminobutylphosphonic acid, ABP, or poly(ethyleneimine), PEI) and carboxylic acid (phosphonoundecanoic acid, PNDA) functionalized monolayer-modified substrates. **(c)** In case of a methyl-terminated monolayer, (tetradecyl phosphate, TDP), no ligand exchange occurs.

FePt NPs were assembled on modified Al_2_O_3_ substrates in two steps. Activation of the substrate was induced by a coupling layer, followed by ligand exchange between the surfactants around the NPs and functional groups of the adsorbate on the modified Al_2_O_3_ substrates. The NPs are stabilized with the surfactants oleic acid and oleyl amine (Figure 1). Oleyl amine binds to Pt through the amino group and oleic acid binds to Fe through the carboxylic acid group [9]. They can be replaced by other acids or amines, or by surfactants with a higher affinity to either Fe or Pt [9]. Thus, adsorbates with terminal amine or carboxylic acid functional groups were chosen (Figure 1). PEI and [3-(2-aminoethylamino)propyl]trimethoxysilane have been used before for binding of particles through ligand exchange [1,10,13].

### 2.1. FePt Nanoparticles

**Figure 2.**
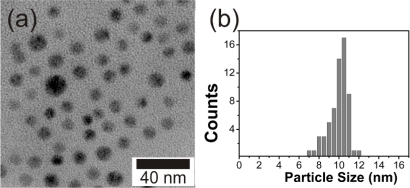
**(a)** TEM image of FePt NPs. **(b)** Histogram of FePt NPs stabilized with oleyl amine and oleic acid.

FePt NPs were prepared by reduction of Pt(acac)_2_ and decomposition of iron pentacarbonyl in the presence of oleyl amine and oleic acid surfactants, followed by precipitation of the NPs by using ethanol and redispersion in hexane. A drop of a solution of the NPs in hexane was deposited on a carbon-coated copper grid for TEM analysis. The particle size was determined to be 10 ± 2.3 nm as shown in Figure 2.

To analyze the elemental composition of the NPs, a PEI-covered Al_2_O_3_ substrate was immersed into a FePt solution to bind the FePt NPs. After evaporation of the solvent, the sample was analyzed by X-ray photoelectron spectroscopy (XPS), which showed an elemental composition of Fe_0.58_Pt_0.42_. Due to PEI, C and N were also detected. The ratio of Fe:Pt is close to 1:1, as expected from the synthesis procedure [1].

### 2.2. SAM Formation

The preparation and characterization of monolayer-modified Al_2_O_3_ substrates was performed according to literature procedures [9,25,31,35,36,38–40]. Clean Al_2_O_3_ substrates were immersed into ABP, PNDA or TDP solutions for two days at room temperature, rinsed afterwards with solvent and dried under a flow of N_2_ to yield amino, carboxylic acid and methyl-functionalized substrates, respectively. Clean Al_2_O_3_ substrates were immersed into a PEI solution for five minutes and then dipped in ethanol several times to wash off excess PEI to yield amino-functionalized substrates. In the case of PEI-covered alumina, AFM results showed that the surface was smooth and homogeneous (Figure S4), and XPS verified the presence of C and N. After scratching the PEI layer with an AFM tip, the measured thickness of the PEI layer was around 3.0 nm. The surface of TDP-functionalized alumina still shows atomic steps (Figure S1), which indicates the SAM layer covers the surface homogeneously. For obtaining information on the thickness of the TDP layer, microcontact printing was applied. As shown below (Figure 4a), the height of the TDP features was around 1.5 nm. This is somewhat lower than the extended adsorbate length (2 nm), which indicates a tilt in the SAM layer similar to various alkylphosph(on)ate SAMs on metal oxides [31,35,40,41]. The water contact angle (CA) of oxygen plasma-cleaned Al_2_O_3_ was below 10°, which increased to 70°, 60° and 115° for SAMs of ABP, PNDA and TDP, respectively. The high CA value (115°) of a TDP SAM indicates a quite hydrophobic surface and this confirms a CH_3_ termination. The CA values of (70° and 60°) are somewhat high for a hydrophylic surface. Zwahlen *et al.* [42] reported that OH terminated dodecylphosphates on TiO_2_ were less ordered than their methyl terminated counterparts, despite their similar molecule densities. XPS measurements proved that all the expected elements were present on the surface for all SAMs and in the expected ratios (Table S1). A shift of −0.81 eV to lower binding energy was observed for the P peaks of TDP on a SAM (134.2 eV) compared to the bulk (135.01 eV). This indicates a charge transfer from substrate to the PO_4_ headgroup during SAM formation [40].

Angle-dependent XPS indicated that binding to the surface occurred through the PO_4_ headgroup in case of TDP and through the PO_3_ headgroup in case of PNDA (Figure S3). The length of ABP was too small to determine the molecular configuration. Fourier transform infrared spectroscopy (FTIR) by scanning a TDP SAM-covered alumina substrate and subtracting the background signal of bare alumina, showed that CH_2_ asymmetric and symmetric stretch vibrations of alkyl chains were around 2919 and 2951 cm^−1^ respectively (Figure S2). This is an indication of the semicrystalline character of the alkyl chains [35,41]. Since binding occurs via the phosph(on)ate headgroup in ABP, PNDA and TDP, similar binding modes were assumed. In conclusion, all the measurements indicate the successful formation of the monolayers on alumina with a high coverage.

### 2.3. Assembly of FePt NPs

**Figure 3.**
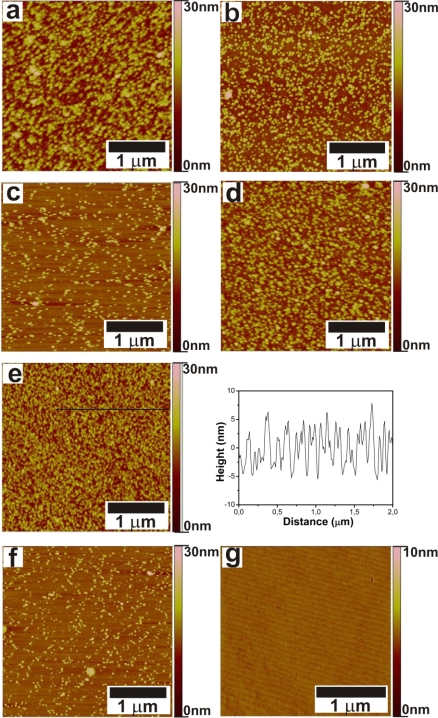
AFM images of FePt NPs assembled on **(a)** PNDA-modified Al_2_O_3_ substrate, 15 min immersion. **(b)** PEI-modified Al_2_O_3_ substrate, 15 min immersion. **(c)** ABP-modified Al_2_O_3_ substrate, 15 min immersion. **(d)** PEI-modified Al_2_O_3_ substrate, 90 min immersion. **(e)** ABP-modified Al_2_O_3_ substrate, 90 min immersion, with section analysis. **(f)** Bare Al_2_O_3_ substrate, after 90 min immersion. **(g)** TDP-modified Al_2_O_3_ substrate, 90 min immersion.

Figure 3 shows the morphologies of the samples after immersion in the FePt NPs solution. Table 1 gives the nanoparticle densities for different surfaces with different immersion times. Figure 3e and a show that NPs are assembled on surfaces with NH_2_ and COOH-terminated SAMs, respectively. The section analysis in Figure 3e shows that the heights of NPs are around 10 nm, which is in good agreement with particle sizes obtained from TEM. The section analyses of other AFM images indicate similar particle sizes (not shown). Figure 3d shows a relatively high coverage and homogeneous distribution of FePt NPs on a PEI-modified Al_2_O_3_ surface. In case of 15 min immersion time, the density of particles on PEI-modified substrates (Figure 3b) is two times higher than the density of the particles at ABP-modified substrates (Figure 3c) as shown in Table 1. PEI, with its branched structure, forms a continuous film on the substrate (Figure S4), and probably exposes more NH_2_ functional groups than an ABP SAM. PEI might have a sloppier packing, thus some chains may stick out to bind the NPs. These resulted in binding of more particles on the PEI-modified surface than on the ABP-modified surface after the same immersion time.

For 10 nm NPs, a maximum coverage of around 40×10^10^ NPs/cm^2^ is expected for random packing (assuming half of hexagonal packing). However, the maximum NP coverage reached is about one twentieth of this value. The organic monolayers have high coverage on the alumina substrate and the low degree of particle adsorption is therefore not due to the SAM layer. An increased NP coverage with time is observed (Figure 3 and Table 1), indicating the process is not yet over after 90 min immersion. The coverage can potentially be further increased by longer immersion times or by increasing the NP concentration. Additionally, some form of surface aggregation might have occurred due to necking of two or more NPs during NP adsorption, which cannot be resolved by AFM. AFM therefore can give only lower limits, and higher coverages have not been attempted here since AFM analysis would have become useless. As shown in Figure 3g, no particles are present on a TDP-modified surface after 90 min immersion. FePt NPs did adsorb on bare Al_2_O_3_ (Figure 3f) but less compared to functionalized surfaces. Of the phosph(on)ate SAMs, PNDA-modified Al_2_O_3_ substrates provide relatively high coverages. The relative rates of binding of NPs on modified surfaces follow ABP<PEI∼PNDA. The difference in binding kinetics between COOH and NH_2_-covered substrates, as between PNDA (Figure 3a) and ABP (Figure 3c) for 15 min immersion, is most likely due to a higher the ligand exchange rate for COOH groups.

**Figure 4.**
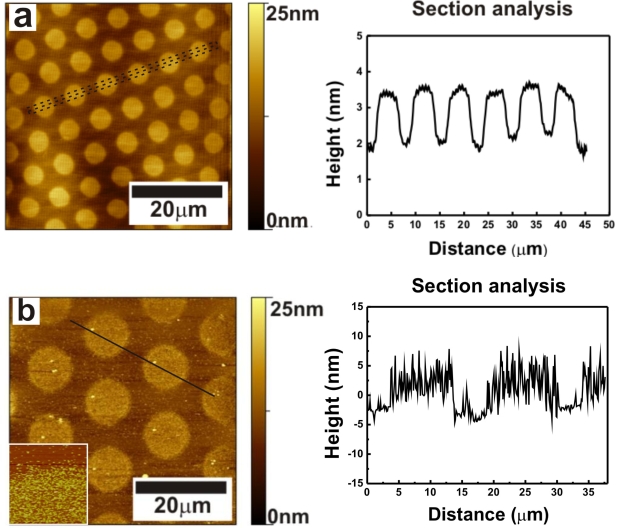
AFM images of **(a)** TDP patterns on alumina prepared by microcontact printing. **(b)** FePt NPs assembled onto an Al_2_O_3_ substrate patterned with ABP by microcontact printing, inset is a 3x3 μ^2^ AFM image of the same sample at the pattern boundary.

Figure 4a shows an AFM image of a patterned alumina surface prepared by microcontact printing (μCP), using TDP as an ink. From the height image, it is clear that a well defined, uniformly distributed TDP pattern is formed on the Al_2_O_3_ surface. ABP was also printed in a similar way but the height of the ABP molecules was too small to provide a clear contrast in the height image. However, friction imaging showed the presence of the pattern (Figure S5).

The affinity contrast between bare and ABP-modified alumina was employed further to create FePt patterns on the substrate. Printed ABP-patterned alumina substrates were immersed in a FePt solution for 120 min. As seen in Figure 4b, there is a clear contrast between ABP-covered regions and bare parts due to the preferential assembly of the FePt NPs on the NH_2_-terminated areas. NP density at the printed region is (1.92±0.1)×10^10^ NPs/cm^2^, similar to the value for ABP-modified Al_2_O_3_ substrate with 90 min immersion, and at the non-printed region the density is (0.44±0.02)×10^10^ NPs/cm^2^. This shows that microcontact printing is an efficient tool to create patterns of FePt NPs by directed assembly.

**Figure 5.**
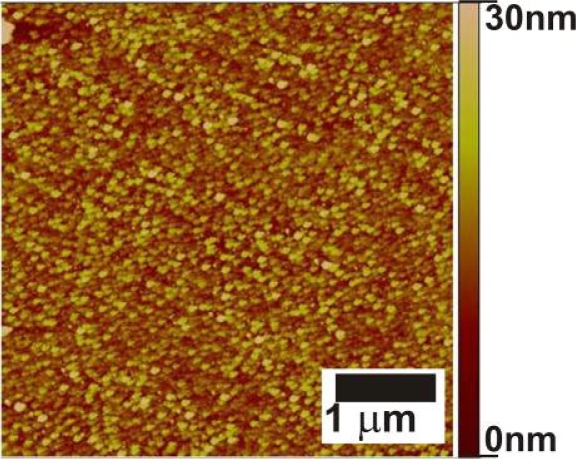
AFM image of FePt NPs assembly (90 min) on an ABP-modified Al_2_O_3_ substrate, after annealing under reducing environment (96%N_2_/4%H_2_) for 1 h at 800 °C.

Figure 5 shows that individual NPs can still be distinguished after thermal annealing. The apparent NP density after annealing is (1.27±0.06)×10^10^ NPs/cm^2^ which is lower than the value before annealing which may indicate a certain degree of aggregation. Aggregation of the particles upon annealing is a common problem and the use of linkers to anchor the particles partly prevents this [9]. Yu et. al. [13] have shown that a self-assembled [3-2-aminoethylamino)propyl]trimethoxysilane monolayer was effective to stabilize the FePt NPs on SiO_2_ surfaces and to prevent coalescence of particles upon annealing.

### 2.4. Structural and Magnetic Properties

**Figure 6.**
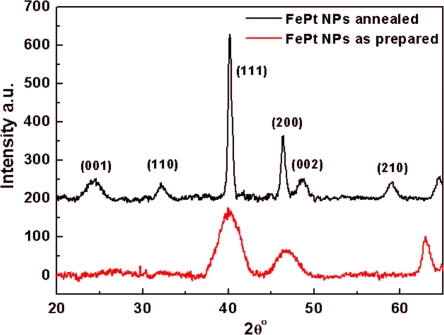
XRD patterns of FePt NPs on a glass substrate before (red) and after (black) annealing for 1 h at 800 °C.

To investigate the effect of thermal annealing on the particle crystallinity, a thick layer of FePt NPs was prepared by casting a 20 mg/ml FePt solution on a glass substrate followed by evaporation of the solvent without rinsing and by using the same annealing procedure as described above. Figure 6 shows the XRD patterns of the FePt multilayers before and after annealing for 1 h at 800 °C. It shows the evolution of the superlattice peaks (001) and (110), as well as the fundamental peak (002), which indicates the transformation of the lattice from FCC to FCT (L1_0_) [43–45].

**Figure 7.**
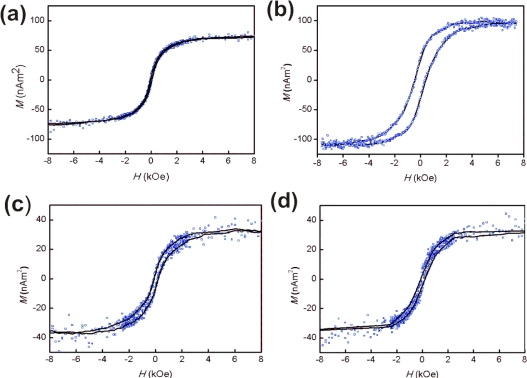
In-plane field hysteresis loops of FePt NPs assembled on **(a)** PEI-modified Al_2_O_3_ before annealing. **(b)** PEI-modified Al_2_O_3_ substrate after annealing. **(c)** ABP-modified Al_2_O_3_ substrate after annealing. **(d)** PNDA-modified Al_2_O_3_ substrate after annealing. Samples were annealed under reducing conditions (96%N_2_/4%H_2_) for 1 h at 800 °C

The magnetic properties of the NPs are related to the crystal structure of the material. To study the effect of phase change upon annealing on the magnetic properties, vibrating sample magnetometer (VSM) measurements were performed. VSM measurements of the annealed FePt NPs on monolayer-covered substrates indicate the distinct ferromagnetic behavior of the NPs at room temperature (Figure 7). Upon annealing, all samples showed a considerable increase in coercivity to 200–450 Oe. This shows that the magnetic properties are not related to the type of chemical functionality on the alumina substrates. The values are small compared to reported coercivity values [1,13,43]. This may be due to an incomplete phase transformation of the nanoparticles, which might be improved by extension of the annealing time. On the other hand, Skomski et. al.[46] have reported that the decrease of the coercivity for FePt NPs with large particle sizes (> 10 nm) is of micromagnetic origin, associated with structural imperfections such as polycrystallinity and reduced anisotropy at the surface.

For a densely packed monolayer, the expected saturation magnetization *M*, which is related to the volume of FePt, is around 120 nAm^2^ based on the momentum density of bulk FePt (1140 emu/cc [24]). The measured intensity of *M* values for the samples are below this value (Table 1, Figure 7) which shows that the coverage is on the order of magnitude of a monolayer. Thus, VSM results indicate the actual coverage would be higher than calculated by counting the NPs from AFM images, indicating some degree of aggregation.

## 3. Experimental Section

### 3.1. Materials

Polished substrates of R-(1–102) Al_2_O_3_ (1×10×10 mm) were purchased from SurfaceNet GmbH, Germany. These substrates were cut into 5×5 mm^2^ pieces with a diamond saw and cleaned by ultrasonication in acetone and ethanol for 30 min each. Tetradecylphosphoric acid (TDP) was supplied by A. Wagenaar and J. Engbersen (RUG, Groningen). Aminobutylphosphonic acid (ABP, purity 99%), phosphonoundecanoic acid (PNDA, purity 96%), poly(ethyleneimine) (PEI), Pt(acac)_2_ and oleic acid were purchased from Sigma-Aldrich. Oleyl amine was purchased from Fluka. Hexadecanediol and iron pentacarbonyl were purchased from ABCR.

### 3.2. Synthesis of FePt NPs

Monolayer-protected FePt NPs were synthesized via a modified method reported by *Sun et al.* [1]. A solution of 0.25 mmol Pt(acac)_2_ and 0.75 mmol 1,2-hexadecanediol in 20 mL octyl ether was heated to 80° C, and to this solution 0.5 mmol oleic acid, 0.5 mmol oleyl amine and 0.5 mmol Fe(CO)_5_ were added via a syringe under a fume hood. Caution: the decomposition of Fe(CO)_5_ produces CO, which is potentially lethal. The mixture was further heated to 150°C for 1 h. The black product was precipitated using ethanol and the particles were redispersed in hexane. This procedure was reported to yield a 1:1 Fe:Pt ratio in the NPs [1,9].

### 3.3. Sample Preparation

Oxygen plasma cleaned Al_2_O_3_ substrates were immersed into 1 mM ABP solution in 100:1 v/v hexane:isopropanol, a 1 mM PNDA solution in 50:50 v/v ethanol:H_2_O, or a 0.125 mM TDP solution in 100:1 v/v hexane:isopropanol for two days at room temperature. Afterwards, the samples were rinsed with the corresponding pure solvents or solvent mixtures, and dried under a flow of N_2_. In the case of PEI, clean substrates were immersed in a 20 mg/ml PEI solution in chloroform for five min and then dipped in ethanol several times to wash off excess PEI.

### 3.4. Microcontact Printing

Silicon masters with micrometer-sized features were fabricated by photolithography. PDMS stamps were prepared from commercially available Sylgard-184 poly(dimethylsiloxane) (Dow Corning). The curing agent and the prepolymer were manually mixed 1:10 volume ratio and cured overnight at 60 °C against the master. The cured stamp was peeled off from the master at the curing temperature. Before printing, the stamps were rinsed with pure ethanol and dried under a flow of N_2_. The stamps were inked with a few drops of solutions of TDP in ethanol or ABP in water. For ABP, an oxidized stamp was used [47,48]. The stamps were dried with N_2_ and brought into conformal contact with alumina substrates for five min. After removing the stamps, the samples were rinsed with ethanol to wash off excess ink followed by drying under nitrogen.

### 3.5. Nanoparticle Assembly

Al_2_O_3_ substrates covered with a self-assembled monolayer (SAM) of TDP, ABP, PNDA, a thin layer of PEI or with a printed ABP pattern were immersed into a FePt (1 mg/ml) solution for 15–120 min to assemble FePt NPs on the modified Al_2_O_3_ surfaces. As a control experiment, a bare alumina substrate was also immersed into the FePt solution for 90 min. Subsequently, the samples were rinsed with pure hexane to wash off physisorbed particles and imaged by AFM.

### 3.6. Thermal Annealing

To obtain the chemically ordered face-centered tetragonal (FCT) L1_0_ phase, which results in ferromagnetic behavior at room temperature, the FePt-covered substrates were annealed in a reducing environment (96%N_2_/4%H_2_) for 1 h at 800 °C.

### 3.7. Measurements

*Atomic force microscopy (AFM):* The morphology of the nanoparticle-covered surfaces was observed by a digital multimode Nanoscope III (Digital Instruments, Santa Barbara, CA) scanning force microscope, equipped with a J-scanner. All measurements were done at ambient in tapping mode or contact mode.

The approximate nanoparticle densities were calculated by counting particles at a certain area. For instance, in the case of assembly on a PEI modified Al_2_O_3_ substrate with 90 min immersion time, counting was done on the AFM image at three different areas, the average densities were calculated, and a standard deviation of 5% was found, which was assumed similar for the other samples.

*Vibrating Sample Magnetometer (VSM):* Magnetic studies were carried out using a DMS Vibrating Sample Magnetometer (model VSM10) with fields up to 1500 kA/m and a sensitivity of 10^−6^ mAm^2^. Measurements were done on NP assemblies on ABP, PNDA and PEI-modified Al_2_O_3_ substrates.

*X-Ray Diffractometry (XRD):* The nanoparticle samples after annealing were analyzed by powder XRD analysis using a Philips X’Pert diffractometer (CuK_α_λ = 1.5418 Å).

*X-Ray Photoelectron Spectroscopy (XPS):* Elemental composition was analyzed by a Physical Electronics Quantera Scanning X-ray Multiprobe instrument, equipped with a monochromatic Al Kα X-ray source operated at 1486.7 eV and 25 W. Spectra were referenced to the main C1s peak at 284.80 eV.

*Fourier Transform Infrared Spectroscopy (FTIR)*: Reflection-FTIR spectra of 1024 scans at 4 cm^−1^ were obtained using a BioRad FTS-60A spectrometer with a liquid nitrogen-cooled cryogenic mercury cadmium telluride detector and RAS accessory (BIO-RAD).

*Contact Angle (CA)*: Measurements were done with a Kruss G10 goniometer equipped with a CCD camera. Contact angles were determined automatically during growth of the droplet by a drop shape analysis. Milli-Q water (18.4 MOhm.cm) was used as a probe liquid.

*High-Resolution Transmission Electron Microscopy (HRTEM):* Particle sizes were analyzed by TEM (Philips CM-30 Twin operating at 200 kV voltage). A drop of NP solution in hexane was deposited on a carbon-coated copper grid.

## 4. Conclusions

The NP coverage on Al_2_O_3_ substrates modified with organic monolayers can be controlled by varying the immersion time into a FePt NPs solution. FePt NPs assemble on ABP, PNDA and PEI SAMs, which have NH_2_ or COOH functionalities, probably by ligand exchange. This gives the possibility to control the adhesion of NPs on surfaces by changing the surface chemistry. The assembly process results in moderately packed FePt monolayers on SAM-covered Al_2_O_3_ substrates. Microcontact printing provides the possibility to direct the NP assembly to designated areas of the substrate. Thermal annealing provides phase transition of FePt NPs which results in ferromagnetic behavior at RT. To prevent non-specific adsorption of the NPs on bare substrate regions, making patterned and backfilled monolayers by two types of SAMs may be a suitable way. The here developed process may be used in the fabrication of spintronic devices.

## Supplementary Information

### Preparation and Characterization of SAMs on Al_2_O_3_

In Figure S1 atomic force microscopy (AFM) images of SAM-functionalized and bare alumina surfaces are shown. As clearly seen in Figure S1b, thermal annealing of the substrates before SAM formation resulted in sharp step edges and smoother surfaces [1,2] when compared to Figure S1a which shows a bare alumina surface without annealing. Figure S1d shows a TDP SAM on annealed alumina, which looks quite similar to the annealed bare Al_2_O_3_ surface, indicating a homogeneous coverage. Sharp step edges and wide and smooth steps are clearly seen. The step structure is also visible in case of ABP and PNDA SAMs which have amino and carboxylic acid endgroups, respectively (Figure S1e,f). In all images the step heights are around 0.3 nm. Since AFM is sensitive to height differences with atomic resolution in the vertical direction, the organic layers must have assembled on the surface with a homogeneous thickness.

Figure S1. Contact (CM) and tapping mode (TM) AFM height images of blank and SAM-functionalized Al_2_O_3_ surfaces (a) blank (CM), (b) blank, annealed at 1000 °C for 2 h (T.M.), (c) TDP SAM on blank (CM), (d) TDP SAM on annealed alumina (T.M), (e) ABP SAM on blank (CM), (f) PNDA SAM on blank (CM).

In Figure S2, a Fourier-transform infrared spectrum (FTIR) of a TDP SAM on alumina is shown.

Figure S2. FTIR of TDP SAM on Al_2_O_3_ substrate.

In Table S1 the results for X-ray photoelectron spectroscopy (XPS) analysis of SAMs on alumina is shown. XPS results show good correlation with the expected ratios of the elements, with one exception for ABP, probably due to carbon contamination.

**Table S1.** Selected XPS Data of SAMs on Al_2_O_3_ substrate.

SAM	C/P (XPS)	C/P (calcd)	N/P (XPS)	N/P (calcd)
TDP (C _14_PO _4_)	15.60±0.59	14	-	-
PNDA(C _11_PO _5_)	11.76±0.7 2	11	-	-
ABP (C _4_NPO _3_)	13.760±50	4	0.81±0.12	1

To observe the orientation of TDP and PNDA molecules, angle-dependent-XPS was done and the results are shown in Figure S3a and S3b. The electron takeoff angles varied between 5–90 ° (angle values are relative to the surface plane). The results show a clear dependence of the elemental peak intensities on the detection angle. As the detection angle increases, the amount of C1s from the alkyl chain decreases and the contribution of P from the headgroup increases. This indicates that P is located in the inner part of the SAM which is closer to the substrate surface when compared to C [3]. The result is in line with the CA values which suggests tails-up orientation.

To obtain information on the configuration of the TDP SAM, the monolayer was sputtered with Ar ions sequentially within the XPS chamber and the XPS analyses were done after each sputtering. From Figure S3c the gradual decrease of the C content is observed upon sputtering, while P remains on the surface. This is another indication that P is closer to the alumina surface. Angle-dependentXPS and sputtering showed that C was on top of P in case of PNDA and TDP. The results are in line with the literature, since alkylphosphates and alkylphosphonates were reported to bind metal oxides through the phosph(on)ate headgroup [3–5].

Figure S3. Angle-dependent XPS of SAM-covered Al_2_O_3_ substrates of (a) TDP and (b) PNDA; (c) XPS on TDP SAM on Al_2_O_3_, using sputtering for depth profiling.

Figure S4. AFM image of (a) PEI-modified alumina (TM), (b) PEI scratched by AFM tip (CM).

Figure S5. AFM image of ABP patterns on alumina prepared by microcontact printing, friction mode.

References1.MesserschmidtCSchwartzDKGrowth mechanisms of octadecylphosphonic acid self-assembled monolayers on sapphire (corundum): Evidence for a quasi-equilibrium triple pointLangmuir2001174624672.YoshimotoMMaedaTOhnishiTKoinumaHIshiyamaOShinoharaMKuboMMiuraRMiyamotoAAtomic-Scale Formation Of Ultrasmooth Surfaces On Sapphire Substrates For High-Quality Thin-Film FabricationAppl. Phys. Lett199567261526173.TextorMRuizLHoferRRossiAFeldmanKHahnerGSpencerNDStructural chemistry of self-assembled monolayers of octadecylphosphoric acid on tantalum oxide surfacesLangmuir200016325732714.GawaltESAvaltroniMJKochNSchwartzJSelf-Assembly and bonding of alkanephosphonic acids on the native oxide surface of titaniumLangmuir200117573657385.SporiDMVenkataramanNVTosattiSGPDurmazFSpencerNDZurcherSInfluence of alkyl chain length on phosphate self-assembled monolayersLangmuir200723805380601756954910.1021/la700474v

## Figures and Tables

**Table 1.** FePt NP densities on modified alumina surfaces.

Surface modification	Immersion time (min)	NP density x10^10^ (NPs/cm^2^)	Saturation magnetization M (nAm^2^)
PNDA	15	1.8±0.1	32
PEI	15	1.8±0.1	n.m
ABP	15	1.0±0.05	n.m
PEI	90	2.0±0.1	100
ABP	90	2.2±0.1	32
Bare Al_2_O_3_	90	0.8±0.04	n.m
TDP	90	0.0	n.m

* n.m.: not measured
