# Supplementary figures and images for: Monolayer-directed Assembly and Magnetic Properties of FePt Nanoparticles on Patterned Aluminum Oxide
Source: Int J Mol Sci. 2010 Mar 19;11(3):1162–79. doi: 10.3390/iijms11031162 (PMC2869229; doi:10.3390/iijms11031162)

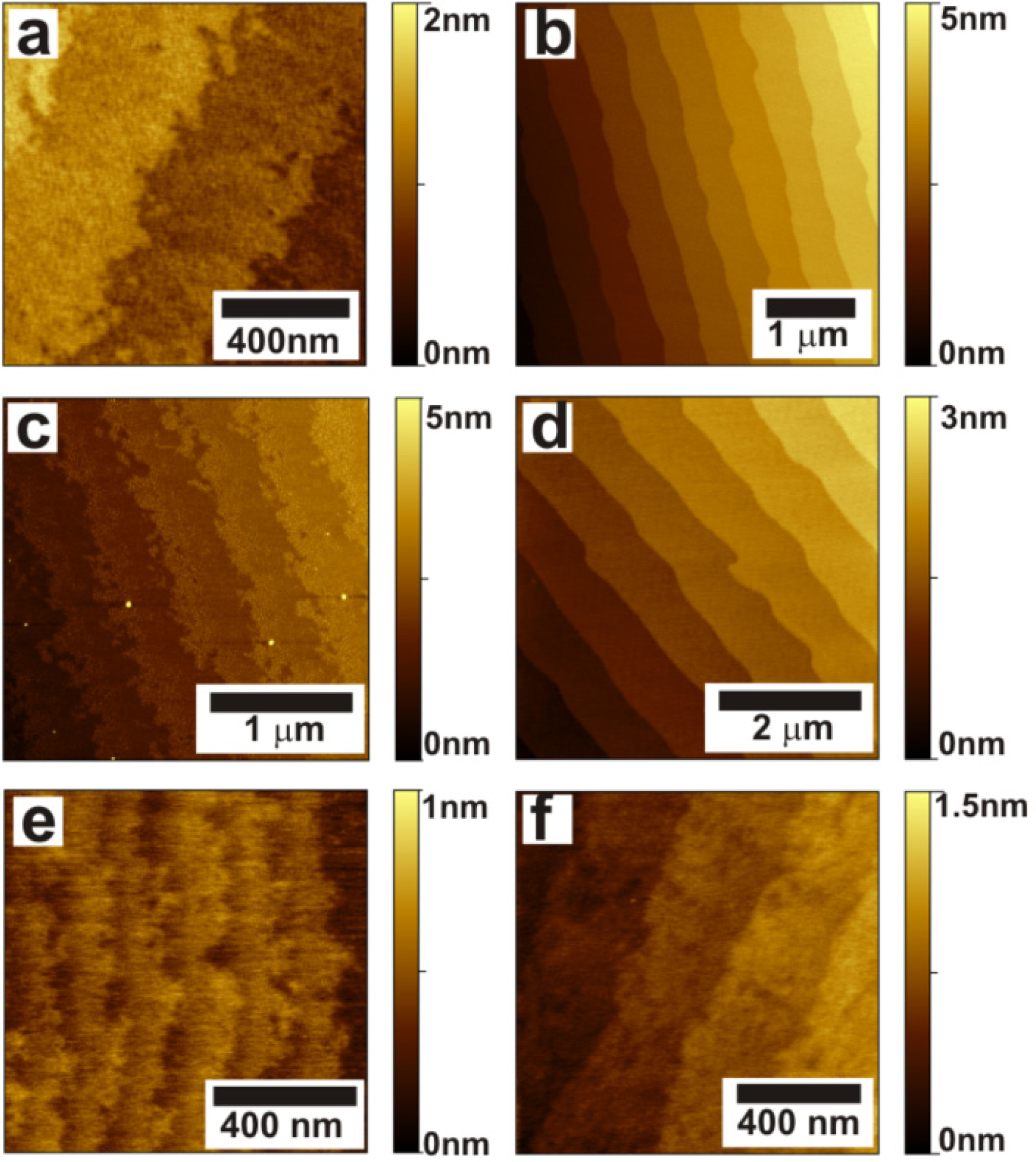

Supplement: Figure S1. — Contact (CM) and tapping mode (TM) AFM height images of blank and SAM-functionalized Al2O3 surfaces (a) blank (CM), (b) blank, annealed at 1000 °C for 2 h (T.M.), (c) TDP SAM on blank (CM), (d) TDP SAM on annealed alumina (T.M), (e) ABP SAM on blank (CM), (f) PNDA SAM on blank (CM). [file ijms-11-01162f8.tif]

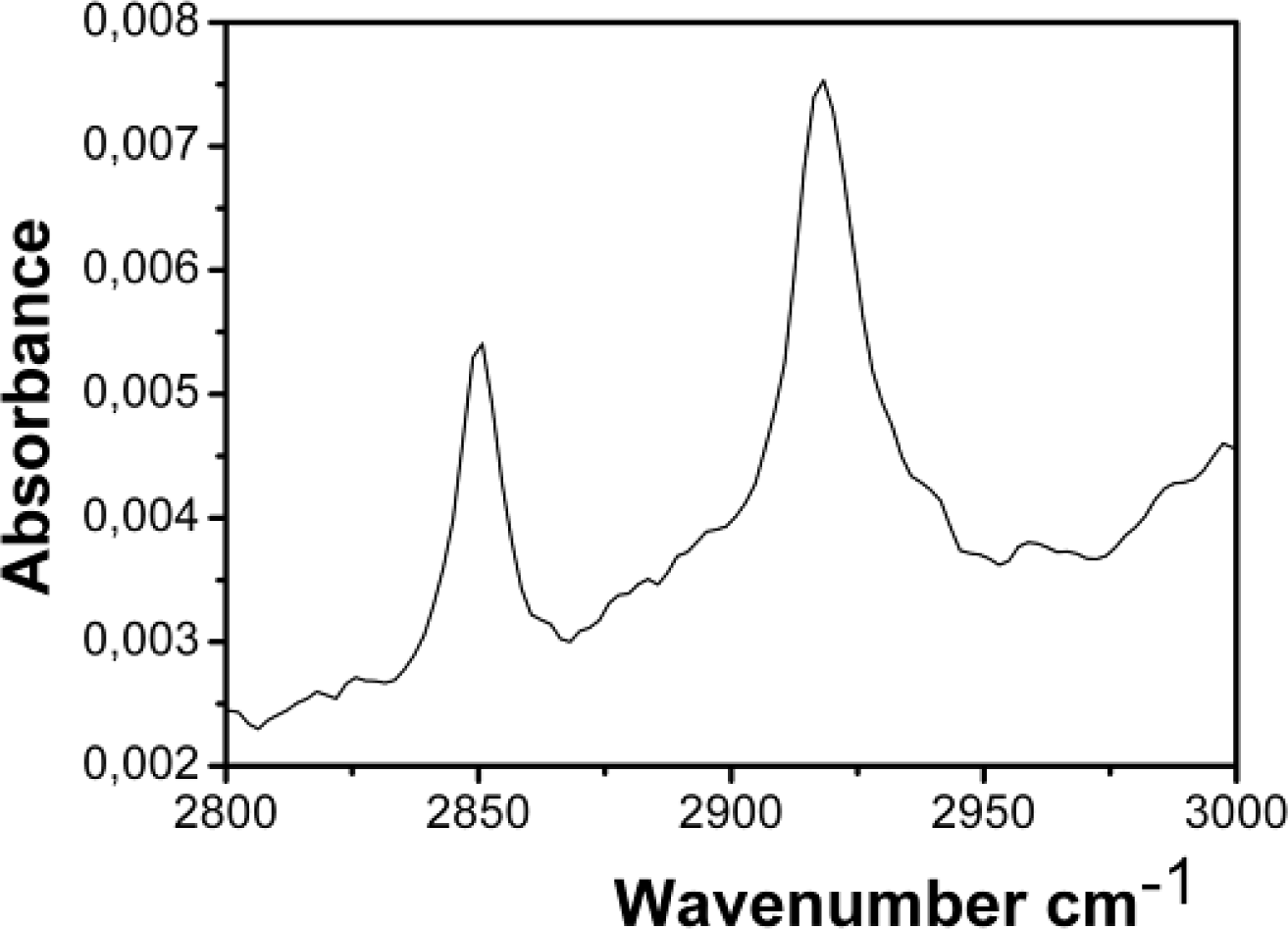

Supplement: Figure S2. — FTIR of TDP SAM on Al2O3 substrate. [file ijms-11-01162f9.tif]

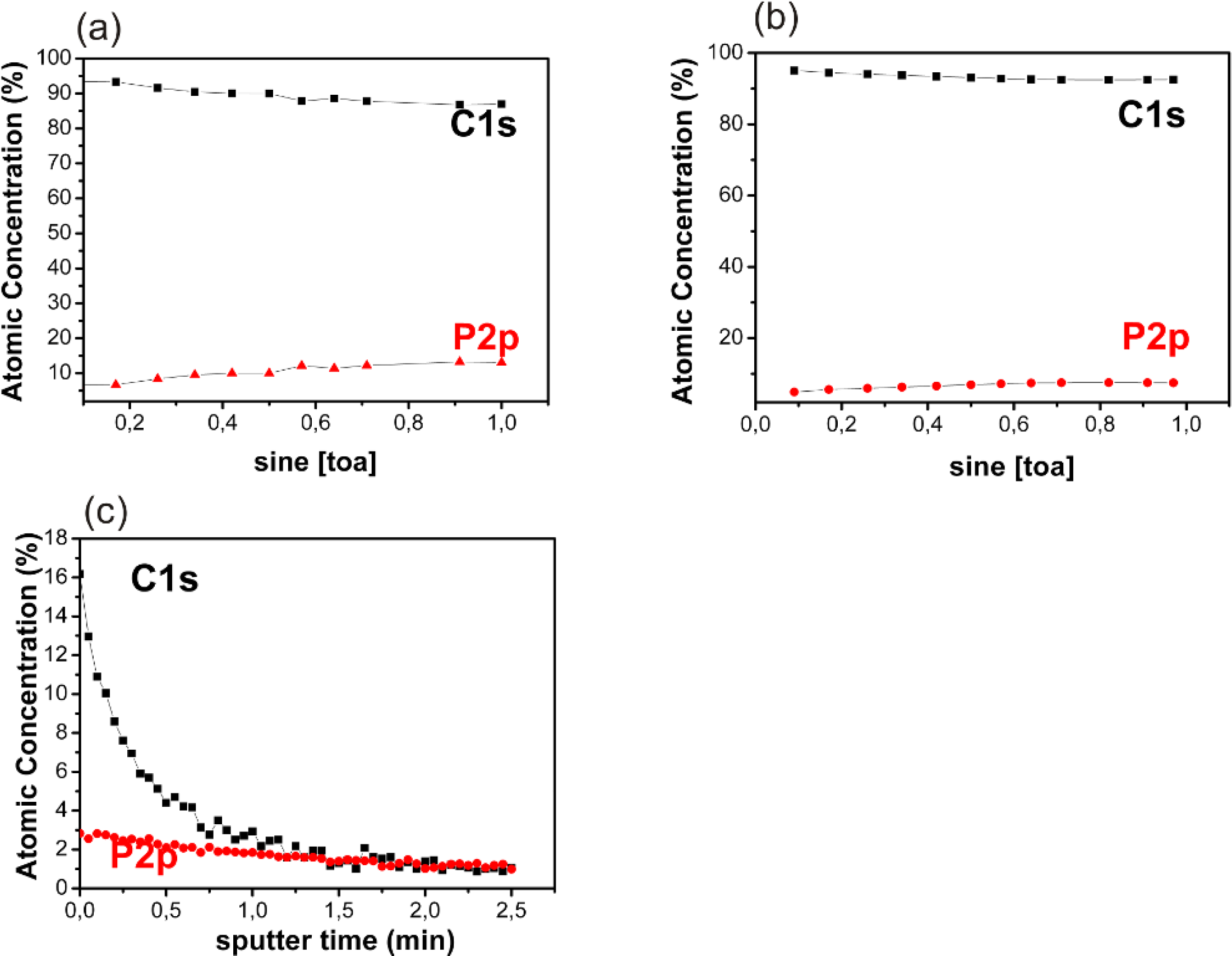

Supplement: Figure S3. — Angle-dependent XPS of SAM-covered Al2O3 substrates of (a) TDP and (b) PNDA; (c) XPS on TDP SAM on Al2O3, using sputtering for depth profiling. [file ijms-11-01162f10.tif]

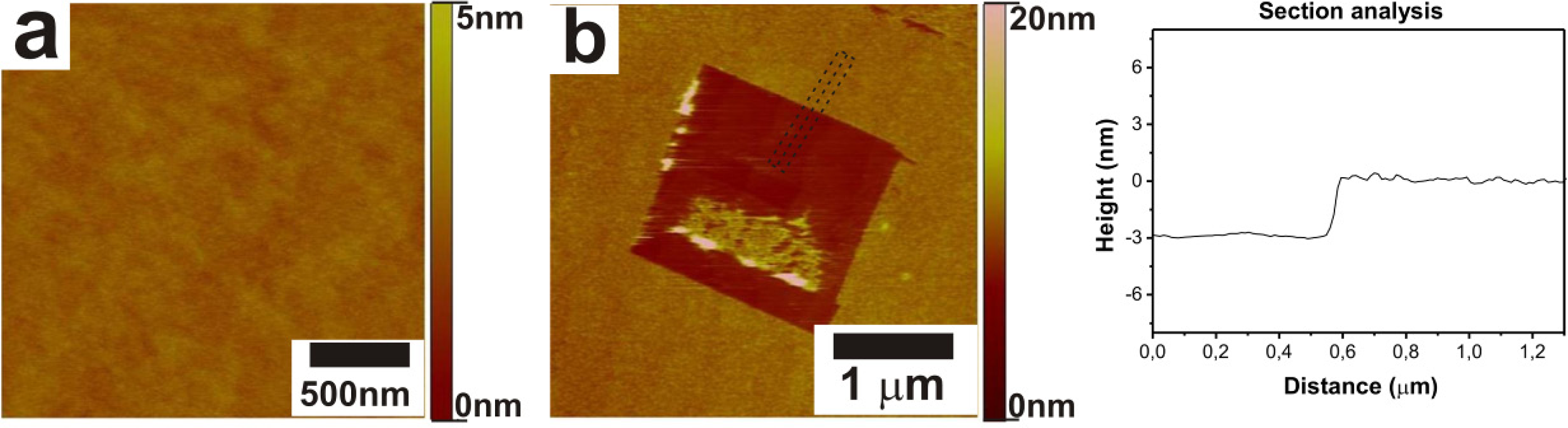

Supplement: Figure S4. — AFM image of (a) PEI-modified alumina (TM), (b) PEI scratched by AFM tip (CM). [file ijms-11-01162f11.tif]

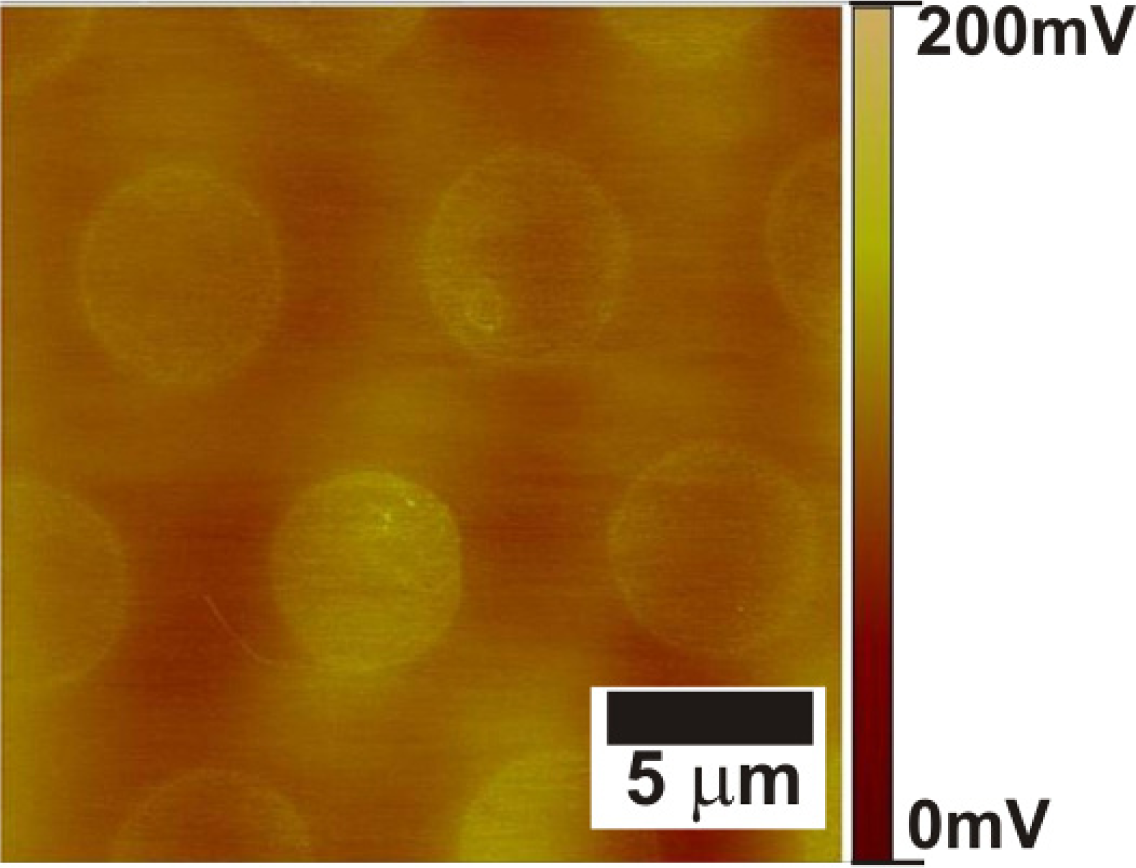

Supplement: Figure S5. — AFM image of ABP patterns on alumina prepared by microcontact printing, friction mode. [file ijms-11-01162f12.tif]
